# Experimental and Numerical Study on Tensile Strength of Concrete under Different Strain Rates

**DOI:** 10.1155/2014/173531

**Published:** 2014-04-16

**Authors:** Fanlu Min, Zhanhu Yao, Teng Jiang

**Affiliations:** ^1^College of Mechanics and Materials, Hohai University, 1 Xikang Road, Nanjing 210098, China; ^2^State Key Laboratory for Geomechanics and Deep Underground Engineering, China University of Mining and Technology, Xuzhou 221116, China; ^3^CCCC Tunnel Engineering Company Limited, Beijing 100088, China; ^4^Geotechnical Research Institute of Hohai University, Nanjing 210098, China

## Abstract

The dynamic characterization of concrete is fundamental to understand the material behavior in case of heavy earthquakes and dynamic events. The implementation of material constitutive law is of capital importance for the numerical simulation of the dynamic processes as those caused by earthquakes. Splitting tensile concrete specimens were tested at strain rates of 10^−7^ s^−1^ to 10^−4^ s^−1^ in an MTS material test machine. Results of tensile strength versus strain rate are presented and compared with compressive strength and existing models at similar strain rates. Dynamic increase factor versus strain rate curves for tensile strength were also evaluated and discussed. The same tensile data are compared with strength data using a thermodynamic model. Results of the tests show a significant strain rate sensitive behavior, exhibiting dynamic tensile strength increasing with strain rate. In the quasistatic strain rate regime, the existing models often underestimate the experimental results. The thermodynamic theory for the splitting tensile strength of concrete satisfactorily describes the experimental findings of strength as effect of strain rates.

## 1. Introduction

It has long been known that concrete materials have a low tensile strength compared to their compressive strength. Since concrete is inherently weak in tension, it has been used as compressive member material in most concrete structures [1–5]. However, even though static tensile loads on concrete members are avoided, it is difficult to isolate concrete members from dynamic tensile stresses. The propagation of tensile stress wave in structural members is generated by explosives, impingement of projectiles, earthquakes, and so on [6, 7]. In fact, in the Great Hanshin-Awaji Earthquake, some uncommon fractures and damages of concrete structures were observed which might have been caused by the propagation of stress waves and/or interface of tensile stress waves.

At a time when a concrete structure comes under dynamic loading, two different modes of failure should be distinguished: local effects and the global effects on the structure. With the current computational facilities and the knowledge on computational modeling, force and stress distributions can be calculated in concrete structures under complex dynamic loading conditions. However, models for dynamic properties of materials are still in their infancy [8, 9]. Consequently, these material models are the weak link in advanced finite element calculations. Reliable test data, that support modeling, are only available to a limited extent.

The compressive behavior of cement based materials under different strain rates has been studied rather extensively. Results of loading tests have confirmed an increase in compressive strength of concrete subjected to dynamic loading. This general result has been confirmed by many researchers in the course of many decades. Some comprehensive review papers of more recent works on this topic can be found in several surveys. For example, Abrams even in 1917 reported that an increase in the rate of loading was accompanied by an increase in the compressive strength of concrete. The same findings were announced by Wastein [10], and Atchley et al. [11] or Hughes and Watson [12]. But because of difficulties in the test setup and instrumentation, little attempt was made to measure the tensile behavior of cement based materials under dynamic loads and thus little data is available.

From the literature review, it can be found that the dynamic tensile strength has not been studied extensively yet and also the data on the rate effect is mostly in the regime of high strain rates (above 1 s^−1^) [13–15]. The research on moderate and quasistatic strain rates (10^−7^ s^−1^ to 1 s^−1^) is rarely limited.

To investigate the effects of strain rate on the tensile strength of concrete, splitting tensile tests of plain concrete specimens were performed at strain rates between 10^−7^ s^−1^ and 10^−4^ s^−1^ on an MTS material test machine. The primary objective of this study was to develop a method for estimating the tensile strength of concrete at both static and dynamic loading rates. A secondary objective was to gain some insight into failure mechanisms of concrete under different strain rates. Moreover, since tensile strength is an experimentally determined property, it is important to validate experimental results with acceptable numerical and analytical procedures.

## 2. Experimental Procedure

### 2.1. Specimens Preparation

The mix proportion of concrete used is given in Table 1, where Type 42.5R Portland cement was used in all mixes. The mixes contained fly ash to save cement and reduce the heat of hydration for practical application. The crushed granite rock with maximum aggregate size 40 mm was employed as coarse aggregate. The maximum sand grain size was 4 mm. The specific gravities of the fine and coarse aggregates were 2.40 and 2.58, respectively. The coarse aggregate and sand were air-dried prior to mixing.

Following casting, the specimens were covered with a plastic membrane to prevent the moisture from evaporating. Specimens were demolded after 24 hours and moist-cured for 6 months. In this investigation, cubical (150 × 150 × 150 mm) specimens were used. Also, several companion 150 mm cubes were cast for obtaining static compressive and elastic modulus of concrete. The following value was obtained for the concrete at the age of 180 days: compressive strength = 51.8 MPa and elastic modulus = 30.3 GPa.

### 2.2. Splitting Tensile Tests

The tensile strength of concrete can be determined from three types of tests: direct pull tests on briquettes and bobbins, modulus of rupture tests on beams, and splitting tensile tests. There are many technical difficulties in executing a true tensile strength test. A uniform stress distribution which makes it possible to calculate the true tensile strength is difficult to obtain. The method commonly used to determine tensile properties of concrete is the flexural beam test by third-point loading on a beam over a span. The flexural strength is calculated from the bending moment at failure, assuming a straight line stress distribution according to Hooke's law. This is not entirely true; however, the calculated flexural strength may be about twice as high as the true tensile strength. The beam test has the advantage that the end pieces of the broken beam can be used to determine the compressive strength of the concrete. These compressive strength results, however, probably differ more from the actual strength of the field concrete than from the compressive strength based on standard cylindrical specimens.

Many attempts have been made to find a substitute for the beam test, and it is possible that the splitting tensile test of a cylindrical specimen may be the solution to the problem [16]. The splitting tensile strength test method has many merits compared with the direct tensile test method; for example, it can be conducted much more easily, the scatterings of the test results are very narrow, and so on. This method, therefore, has been prescribed in many standards as the standard test method for tensile strength of concrete.

Researchers have indicated that, among the three testing methods (direct tensile, splitting tensile, and flexural tests), the splitting tensile test gives the most accurate measurement of the true tensile strength of concrete-like materials in a wide strain rate [17]. Difficulties are encountered in the direct tensile tests when it requires a pure tensile without eccentricity. Often, when grips are used to anchor the specimen, compression from the grips is combined with tension from testing machine. The particular combination of forces has been shown to result in failure at stress levels below the maximum tensile strength [18].

**Figure 1:**
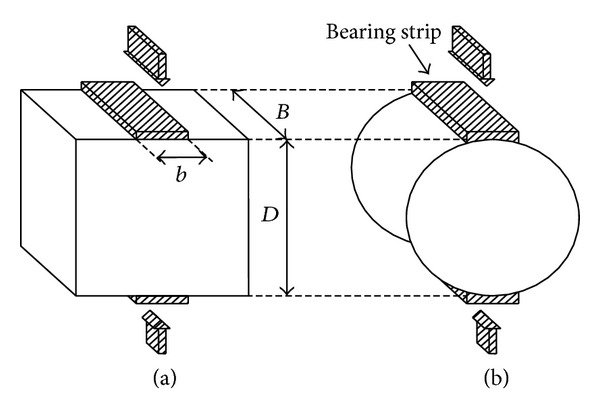
Standard splitting tensile test arrangement.

In the splitting test, a concrete cylindrical or prismatic specimen is compressed along two diametrically opposed generators as shown schematically in Figure 1. A theoretical basis for the test has been postulated by Davies and Bose [19]. The splitting tensile strength is calculated on the assumption of a hypothetical load-bearing strip of zero width (concentrated load).

**Figure 2:**
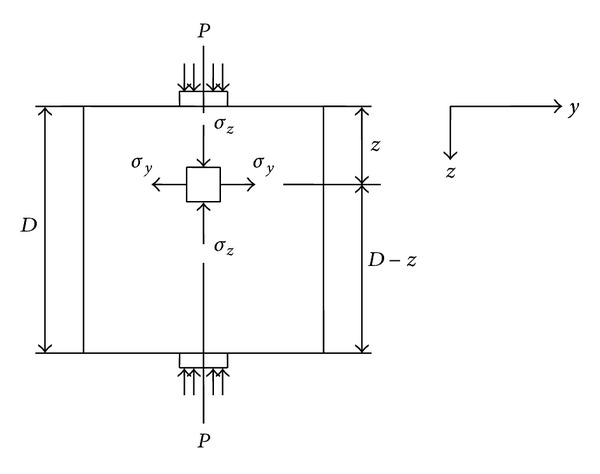
Stresses associated with a splitting tensile test.

The stresses associated with this loading configuration are illustrated in Figure 2. When the compressive load is applied to the specimen, elements located near the center of the cubical specimen along its vertical diameter are subjected to a vertical compressive stress equal to

(1) $$\begin{array}{c} \sigma_{z}=\frac{2P}{\pi BD}\left[\frac{D^{2}}{z\left(D-z\right)}-1\right], \end{array}$$

where *σ*_max⁡,*P*_ is the maximum tensile stress in the specimen when the applied load is *P*, *D* and *B* are the specimen depth and thickness, respectively (Figure 2), and *z* is the distance from the element to the top of the specimen. The element is subjected to a horizontal tensile stress, as well, whose magnitude is equal to

(2) $$\begin{array}{c} \sigma_{y}=\frac{2P}{\pi BD}. \end{array}$$

The narrow bearing strips that are placed between the specimen and the loading platens are used to carry a portion of the high compressive stress that is induced directly beneath the load. The tensile strength determined from test conducted without the bearing strips is typically about 8% lower than the tests conducted with the bearing strips. Although there is a rather high horizontal compressive stress immediately underneath the load, it is accompanied by a vertical compressive stress of comparable magnitude. Therefore, a state of biaxial stress is created, preventing failure in compression [20]. Brittle materials, with a relatively low tensile strength compared to their compressive strength, will tend to fail in tension along the loading line. For each of the splitting tensile experiments, the maximum load was used to calculate the splitting stress at failure (splitting tensile strength) using (2).

Following the standards (Table 2), the maximum tensile stress at failure, calculated from the theory of elasticity, is a material property called splitting tensile strength, *f*_st_. Test made by Thaulow indicated that the splitting strength is largely independent of length and diameter of the specimen. If the load-bearing strips are narrow enough and the material behavior is linear-elastic-brittle, the obtained value is close to the tensile strength determined by an ideal uniaxial tensile test [21, 22]. Finite element analysis has been used to verify that the stress distribution in the splitting tensile sample under dynamic loading is equivalent to that in the static case [23].

**Figure 3:**
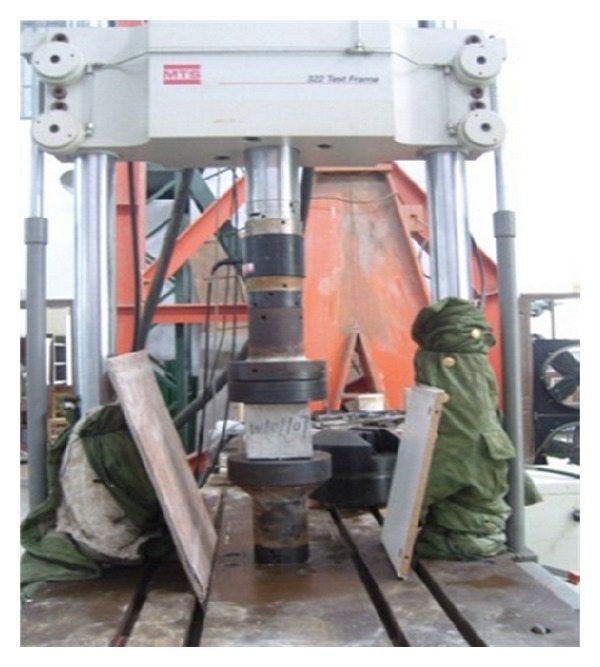
Testing setup for splitting tensile tests.

**Figure 4:**
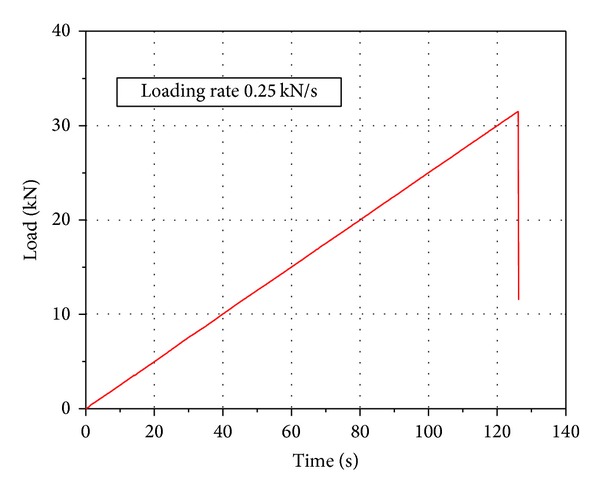
Typical load time curves for concrete: loading rate 0.25 kN/s.

In this study, all strain rate tests were performed using a standard MTS material test machine, as shown in Figure 3. Load was applied at 0.25 kN/s, 2.5 kN/s, 25 kN/s, and 250 kN/s, respectively. The typical loading history is shown in Figure 4. The measurement system consists of a strain amplifier, a tape recorder, and an intelligent signal processor. A 104 Hz sampling frequency can be achieved.

Additionally, the loading rate $\overset{\cdot }{\sigma }$ and the strain rate $\overset{\cdot }{\varepsilon }$ in the specimen can be estimated from the expressions

(3) $$\begin{array}{c} \overset{\cdot }{\sigma }=\frac{f_{\text{td}}}{\tau }, \end{array}$$

(4) $$\begin{array}{c} \overset{\cdot }{\varepsilon }=\frac{\overset{\cdot }{\sigma }}{E}, \end{array}$$

where *τ* is the time lag between the start of loading and the maximum load value (which is determined from the load history present in Figure 4) and *E* is the elastic modulus of the concrete determined from static tests.

## 3. Test Results and Discussion

### 3.1. Crack Pattern and Failure Mode

The failure pattern must guarantee the validity of the expression traditionally used; that is, the rupture must be localized in the diameter coincident with the application of the load [24, 25]. In the tests carried out, same cases had been found. Most of the tests could be classified as “valid tests.” The test results also indicate that splitting tests can be an alternative way to determine the dynamic tensile strength. However, some aspects should be taken into account in future researches. (1) The specimen after failure shows a small broken area near the point of load application. If this observation is confirmed in other cement-based materials, special supports should be designed to avoid any stress concentration in these zones. (2) In this type of tests the material is subjected to a biaxial stress state (tension and compression). It should be important to evaluate the influence of the compression stress in the tensile strength values.

**Figure 5:**
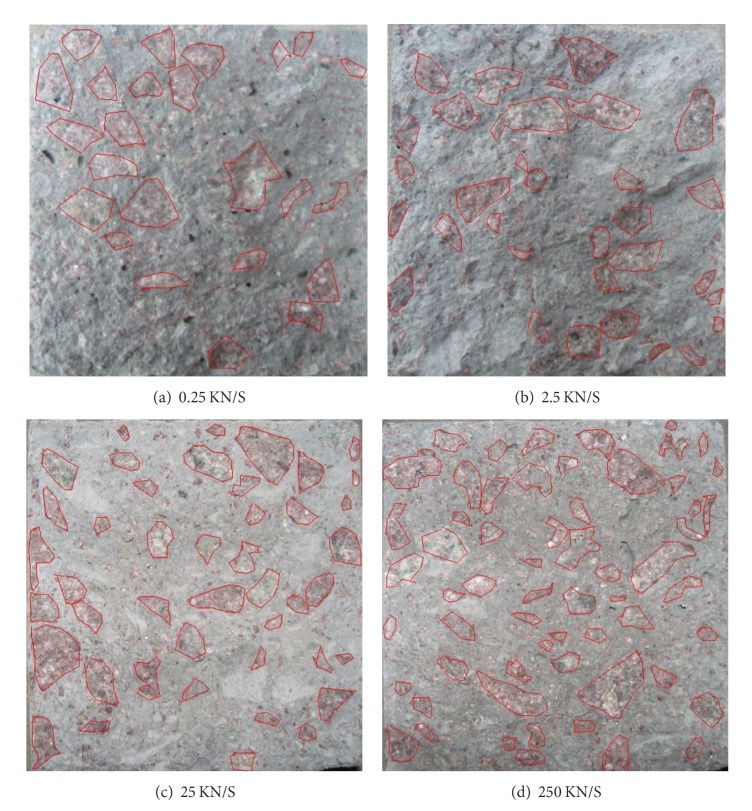
Typical failure surface of concrete beams under typical loading rates.

**Figure 6:**
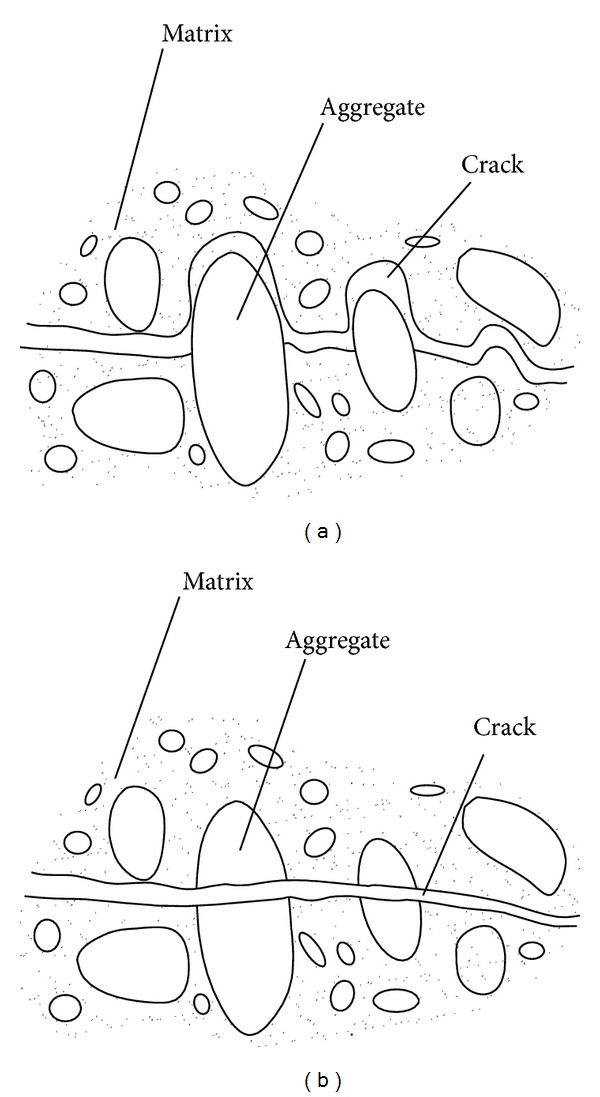
Description of failure (schematic): (a) failure characteristics at static loading rates; (b) failure characteristics at dynamic loading rates.

Figure 5 shows the fractured surfaces of concrete specimens under different loading rates. It can be seen from Figure 6 that the fractured surfaces of the specimens became more and more flattened with the increasing strain rate; and an increasing number of aggregates were broken along the fractured surface. As a consequence of shrinkage effects, microcracks exist in the unloaded concrete mainly at the interface boundaries between the matrix and the aggregates [26, 27]. Upon loading, high stresses occur at the tip of these microcracks. As a result of tensile or compressive strain, these high stresses are relieved by the growth of hair cracks in the matrix and bond cracks at the interface boundaries between the matrix and the aggregates. The material is therefore weakened. Under increasing tensile strain, the specimen stores energy unit total fracture is reached. The stress deformation relationship may be described in two parts. In the first part, which is ascending, the energy gained during loading is not lost upon loading; that is, the energy is reversible. However, in the second, descending part of the stress deformation relationship, a portion of the energy is lost due to crack formation and is therefore irreversible. Both reversible and irreversible energy occur at every point of the descending part of the relationship. Under increasing strain and decreasing stresses these portions decrease.

Generally, at low strain rates in a splitting tensile test, the formation of microcracks is shown after the maximum stress attained. Furthermore, the cracks in the matrix are prevented by the aggregates from growing further. Therefore, they are initially stable; that is, more energy needs to be supplied for their continued propagation. The zone around the microcracks remains capable of carrying load, but in a steadily decreasing amount. This continues until a critical crack width is reached. After this point, the crack is unstable. Finally, the failure occurs at a low reversible energy. The fracture surface grows relatively slowly according to the path of least resistance through the matrix and the interface boundaries around the aggregates; that is, matrix failure occurs (Figure 6(a)). Contrary to the behavior at low strain rates, the resulting stored strain energy remains reversible almost until the maximum stress is reached; this is where energy starts. For delayed crack propagation the cracks occur spontaneously, that is, without significant formation of microcracks. As a consequence, there is a great amount of energy released, the cracks are no longer stable, and they propagate unhindered and relatively quickly. The failure then occurs according to a relatively direct path through the matrix and the aggregates themselves (Figure 6(b)).

### 3.2. Splitting Tensile Strength

Table 3 shows the results for concrete specimens including loading rates, time to failure, strain rates, splitting tensile strength, and dynamic increase factor (DIF).

**Figure 7:**
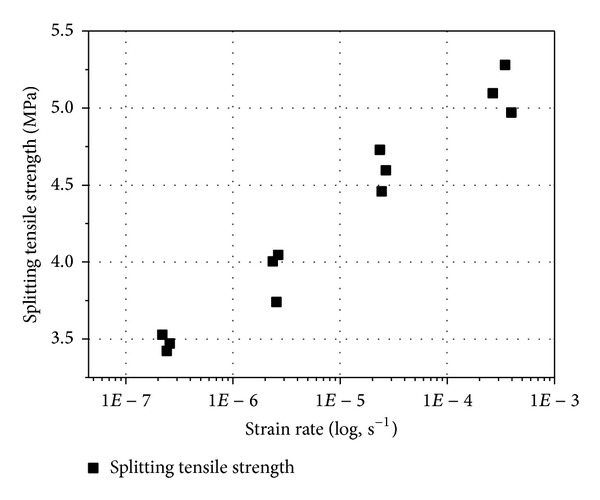
Strain rate dependence of the splitting tensile strength of concrete.

Figure 7 shows the splitting tensile strength of each specimen as a function of strain rate and represents all specimens summarized in Table 3. Figure 7 indicates that, with every order of magnitude increase in strain rate, the splitting tensile strength of concrete increases about 15 percent. This trend is nearly linear increase in strength with each order of magnitude increase in strain rate. On the basis of the results presented here, it is clear that concrete is a very strain rate sensitive material. Concrete beams showed high tensile strength at high strain rate. Several explanations can be suggested to account for these trends. One explanation may be based on fracture mechanics concepts [28, 29]. The phenomenon of strain rate sensitivity can be explained by combining the classical Griffith theory with the concept of subcritical crack growth. According to Griffith's theory, failure in brittle materials occurs when a flaw exceeds the critical flaw size and failure will then occur. If the load is applied very slowly, the subcritical flaws have time to grow and thus the failure occurs at a lower value of load. However, if the load is applied at a very high rate, there is little or no time available for the growth of the subcritical flaws, and a higher load can be reached by the structural element before failure occurs. Zhang et al. [30] reported that prepeak crack growth is reduced at high rates of loading.

An alternative explanation of the observed trend may be given on the basis of nonlinear fracture mechanics. It has been recognized that immediately ahead of a moving crack is a zone of microcracking called the process zone. Wittmann et al. [31] and Reinhardt et al. [32]. have suggested that the size of this zone of microcracking depends upon the velocity of the crack; a faster crack has a larger zone of microcracking ahead of it. At a higher stress rate the crack propagates faster, and therefore the process zone will be bigger. This increased microcracking may explain the higher energy requirements at higher strain rates. This argument may, at first glance, seem to contradict with the argument presented above on the basis of subcritical crack growth, which predicts less microcracking in high strain rate loading situations. However, these two phenomena occur on the opposite sides of the peak load. The concept of subcritical crack growth is applicable prior to the peak load; the concept of a larger process zone is applied in the postpeak load region, where the unstable crack propagation commences.

### 3.3. Dynamic Increase Factor (DIF)

**Figure 8:**
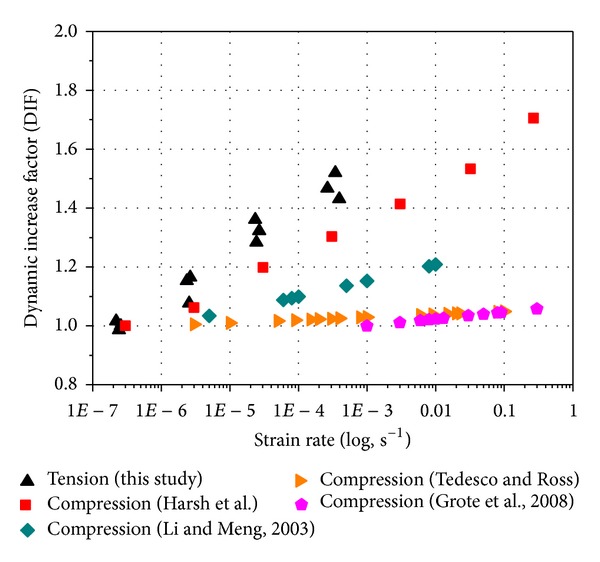
Strain rate dependence of dynamic increase factor (DIF). (Harsh et al. [38], Li and Meng [36], Tedesco and Ross [39], and Grote et al. [35]).

The effect of strain rate on the compressive or tensile strength of concrete-like materials is typically reported as a dynamic increase factor (DIF) (i.e., the ratio of dynamic to static strength) versus strain rate (or the logarithm of strain rate). The use of normalized DIF reduces the influence of the material strength on the DIF formulae [33, 34]. Comparing concrete tensile and compressive strength data from others' literatures [35, 36] versus strain rate (Figure 8), it is apparent that the tensile strength is more sensitive to strain rate effects at lower strain rates than the compressive strength. The same results have been observed by others.

**Figure 9:**
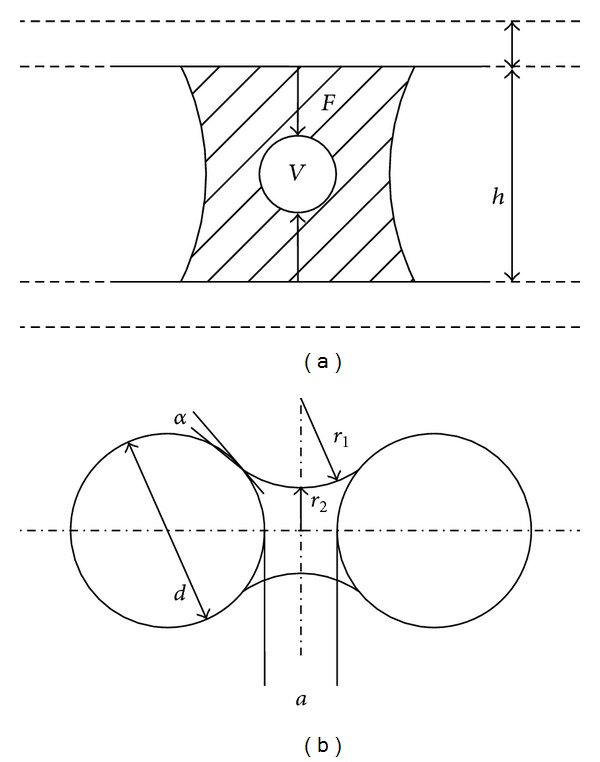
Schematics of Stefan's and capillary effects: (a) Stefan's effect and (b) capillary effect.

The experimental evidence shows also that concrete rate dependence is higher in tension than in compression (Figure 8). In the low to moderate regime, moisture plays an important role in the increase of concrete strength. The free water in the micropores is assumed to exhibit the so-called Stefan effect, causing a strengthening effect in concrete with increasing loading rate. The Stefan effect is the phenomenon that occurs when a viscous liquid is trapped between two plates that are separated quickly, causing a reaction force on the plates that is proportional to the velocity of separation (Figure 9(a)). Candoni et al. [37] claim to give a different explanation of the influence of moisture on the rate effects of concrete. Their interpretation is based on the principle of wave propagation in concrete. When a pore is not filled with water, it will locally reflect the incoming stress wave. The multiple reflections of all pores together can cause a considerable increase in stress. When a stress wave meets a pore that is filled with liquid, the reflected stress is not big enough to cause the increase in stress that locally provokes the damage of the material. However, this interpretation does not explain the increase in strength of concrete between static and dynamic loading.

In authors' opinion, the presence of water in the capillary pores exerts an “external influence” on the material, which results in observed difference in material properties. As the capillary effects (Figure 9(b)), under dynamic loading, the rise of loading rate that resulted from Stefan's effect brings about increase in capillary force, which leads to a compression of solid skeleton which is similar to an “interstressing” of concrete. The external tensile loads should eliminate this inner compressive stress first. Therefore, the Stefan effect will increase the tensile strength. However, under dynamic compressive loading, the increasing external loading rate on the concrete specimen during testing develops an increasing internal pressure, not only on the solid components of the concrete, but also on the liquid in the pores, trying to squeeze the liquid out of the specimen. Since the migration of the liquid is not free due to the smallness of the capillary pore sizes, the hindrance produces a pressure on the contacting pore walls which increases as the external load on the specimen increases. This pore pressure then reduces the magnitude of the external load. Thus, due to the moisture in concrete, the dynamic increase factor (DIF) of compressive strength would be lower than that of tensile strength.

**Figure 10:**
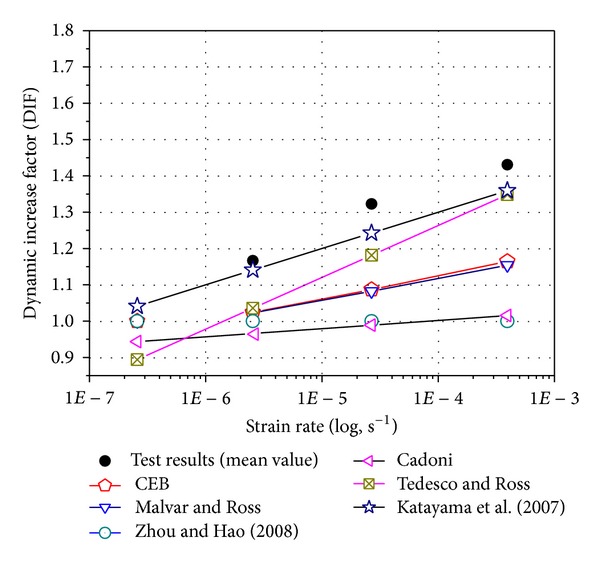
Strain rates influence on the tensile DIF of concrete. (CEB [45], Malver and Ross [41], Zhou and Hao [43], Cadoni [44], Tedesco and Ross [39], and Katayama et al. [42]).

To correctly analyze the influence of the strain rate on the mechanical properties of concrete, the results in terms of failure were elaborated to obtain the DIF, thereby obtaining the curves in Figure 10. Moreover, to verify that the obtained data were consistent with results described in the literature, a comparison with existing empirical models was performed.

The CEB model appears to property fit the available data. The DIF for the tensile strength is given by

(5) $$\begin{array}{c} \text{DIF}=\frac{\sigma_{\text{td}}}{\sigma_{\text{ts}}}=\{\begin{array}{cc} 1, & {\dot{\varepsilon }}_{z}<{\dot{\varepsilon }}_{s},\\ {\left(\frac{{\dot{\varepsilon }}_{z}}{{\dot{\varepsilon }}_{s}}\right)}^{1.016\alpha_{s}}, & {\dot{\varepsilon }}_{s}<{\dot{\varepsilon }}_{z}\leq 30\;{\text{s}}^{-1},\\ {\gamma_{s}\left(\frac{{\dot{\varepsilon }}_{z}}{{\dot{\varepsilon }}_{s}}\right)}^{0.33}, & {\dot{\varepsilon }}_{z}>30\;{\text{s}}^{-1}, \end{array} \end{array}$$

where *σ*_ts_ and *σ*_td_ are the unconfined uniaxial tensile strength in quasistatic and dynamic loading conditions, respectively; *γ*_*s*_ = 10^(7.11*α*_*s*_−1.33)^; *α*_*s*_ = 1/(10 + 6*σ*_cs_/*σ*_co⁡_); ${\overset{\cdot }{\varepsilon }}_{s}=3\;\times \;10^{-6}\;{\text{s}}^{-1}$; *σ*_co⁡_ = 10 MPa; *σ*_cs_ is the unconfined quasistatic uniaxial compressive strength (in MPa).

A series of dynamic splitting tests have been conducted by Tedesco et al. [40]. for concrete specimens with different compressive strengths. Based on the results from these tests, a bilinear tensile DIF regression formula was suggested:

(6) $$\begin{array}{c} \text{DIF}=\frac{\sigma_{\text{td}}}{\sigma_{\text{ts}}}=\{\begin{array}{cc} 1+0.1425\left[\text{lg}\left({\dot{\varepsilon }}_{z}\right)+5.8456\right]\geq 1.0, & \\ \;\;\;\;\;\;\;\;\;{\dot{\varepsilon }}_{z}\leq 2.32{\;\text{s}}^{-1} & \\ 1+2.929\left[\text{lg}\left({\dot{\varepsilon }}_{z}\right)-0.0635\right]\leq 6.0, & \\ \;\;\;\;\;\;\;\;\;\;{\dot{\varepsilon }}_{z}>2.32\;{\text{s}}^{-1}. & \end{array} \end{array}$$

Malvar, and Ross [41]. proposed another formula similar to that of the CEB, which was fitted against the available data for strain rates below 1 s^−1^, and for high strain rates a slope of 1/3 on a log (strain rate) versus log (DIF) was used, also following the CEB formulation. The proposed formulation then becomes

(7) $$\begin{array}{c} \text{DIF}=\frac{\sigma_{\text{td}}}{\sigma_{\text{ts}}}=\{\begin{array}{cc} 1 & {\dot{\varepsilon }}_{z}\leq {\dot{\varepsilon }}_{s}\\ {\left(\frac{{\dot{\varepsilon }}_{z}}{{\dot{\varepsilon }}_{s}}\right)}^{\alpha_{s}} & {\dot{\varepsilon }}_{s}<{\dot{\varepsilon }}_{z}\leq 1{\;\text{s}}^{-1}\\ \gamma_{s}{\left(\frac{{\dot{\varepsilon }}_{z}}{{\dot{\varepsilon }}_{s}}\right)}^{0.33} & {\dot{\varepsilon }}_{z}>1{\;\text{s}}^{-1}, \end{array} \end{array}$$

in which *γ*_*s*_ = 10^(6*α*_*s*_−2)^, *α*_*s*_ = 1/(1 + 8*σ*_*cs*_/*σ*_*co*_), and *ε*_*s*_ = 1 × 10^−6^ s^−1^.

Katayama et al. [42] studied the strain rate effect on the tensile behavior of different concrete. Tests were conducted at the stress rates of 2.5 × 10^−5^ and 8.3 × 10^−5^ N/mm^2^ mm^2^ per millisecond on specimens with different aggregate to cement ratio. They introduced strain rate into Drucker-Prager's equation for the tensile DIF expression for concrete as follows:

(8) $$\begin{array}{c} \text{DIF}=\frac{\sigma_{\text{td}}}{\sigma_{\text{ts}}}=1.0+0.1\left[\text{lg}\left(\frac{{\dot{\varepsilon }}_{z}}{{\dot{\varepsilon }}_{s}}\right)\right]. \end{array}$$

Zhou and Hao [43] recommended a tensile DIF curve for concrete-like materials, which is fitted from experimental results; that is,

(9) DIF=σtdσts={1,ε˙z≤10−4 s−1,1+0.26[lg(ε˙z)+4.0769],10−4<ε˙z≤1 s−1,1+2[lg(ε˙z)+0.53],ε˙z>1 s−1.

Based on the testing results of rocks, Cadoni [44] suggested a tensile DIF formula for concrete aggregates; that is,

(10) DIF=σtdσts={1+0.0225[lg(ε˙z)+5.3333],       ε˙z≤0.1 s−1,1.6+0.7325[lg(ε˙z)]2+1.235lg(ε˙z),        0.1<ε˙z≤50 s−1.

By plotting these relationships against the experimental results (Figure 10), the correspondence with the data obtained can then be appreciated. Moreover, Figure 10 reports DIFs of the tensile strength as evaluated numerically through above expressions (5) to (10). Furthermore, the differences between numerical and experimental values are presented. This shows that, in the quasistatic and moderate strain rate regime, the existing models often underestimate the experimental results. Hence, more suitable expression for designing calculations should be considered.

## 4. Interpretation Test Results with Thermodynamic Model

To treat concrete by thermodynamics means to consider it on an atomic level. Atoms are in a state of continuous motion; attracting and repulsing forces are acting on them. Each atom is situated on a certain energy level. Due to continuous motion there is always a chance that an atom overcomes the inherent energy barrier and moves to another place in the system. If external energy is added to a system of atoms, the energy barrier (activation energy) may be overcome more easily. Energy can be supplied by mechanical loading, heating up, or concentration gradients. The greater these external influences are, the more likely that place changes occur. Place changes of atoms can be detected in an average way by deformations, cracks, or chemical reactions.

After presenting the experimental results, a fracture criterion is formulated to which the data is then compared. This has been done in essentially an empirical manner by combining terms derived from the Arrhenius rate equation to account for the temperature and strain rate effect which was shown to fit the experimental data over the entire range of parameters surprisingly well.

In its simplest form, the rate equation may be written as

(11) $$\begin{array}{c} \dot{\varepsilon }={\dot{\varepsilon }}_{0}\exp\left(-\frac{U\left(\sigma \right)}{RT}\right), \end{array}$$

where the activation energy *U*(*σ*) is assumed to be a function of the effective stress only. For the brittle concrete considered here, fracture is preceded by very little inelastic strain so that it is justified to neglect inelastic strain in the formulation. The other terms in (11) are the absolute temperature *T*, gas constant *R*, and an arbitrary constant ${\dot{\varepsilon }}_{0}$. When applying (11) to the fracture strength, we may consider it in the sense that

(12) $$\begin{array}{c} \dot{\varepsilon }={\dot{\varepsilon }}_{0}\exp\left(-\frac{U\left(\sigma_{f}\right)}{RT}\right), \end{array}$$

where *σ*_*f*_ and *ε*_*f*_ are the stress and strain at fracture. This form is identical to that used by Zhurkov [46] to correlate stress-rupture data for a wide variety of materials including metals, polymers, and glasses. However, in the stress rupture experiment a constant stress is imposed and the time to failure (or creep rate) is measured. In the present tests a constant stress rate is imposed and the resulting stress at failure is measured. Because of the difference in stress history leading to failure, there may not be an equivalence of the constants in correlation equation used. Zhurkov [46] found that the activation energy derived from stress-rupture experiments was nearly equal to the heat of sublimation for many of the materials tested. Based on this observation, he suggested that the actual rupture of interatomic bonds is the controlling mechanism in kinetic fracture of solids.

The experimental data suggests that the stress dependence of the activation energy is linear and of the form

(13) $$\begin{array}{c} U\left(\sigma \right)=U_{0}-\nu \left(\sigma -\sigma_{0}\right), \end{array}$$

where *U*_0_ is the total activation energy of the process, *ν* is a coefficient with dimensions of volume (often called “activation volume”), *σ* is the applied stress, and *σ*_0_ is a constant. (*σ* − *σ*_0_) is the effective stress relative to the thermal activation barrier. The need for inclusion of *σ*_0_ will become evident when examining the data. The linear form of (13) is not essential but is determined from the experimental data itself. Equation (13) could be considered as a two-term truncation of a general Taylor's series expansion of *U*(*σ*).

Substituting (13) in (12) and solving for the applied stress *σ* yield

(14) $$\begin{array}{c} \sigma =\frac{U_{0}}{\nu }+\sigma_{0}-\frac{RT}{\nu }\ln\frac{{\dot{\varepsilon }}_{0}}{\dot{\varepsilon }}. \end{array}$$

It will be noted that *U*_0_/*ν* + *σ*_0_ is the limiting stress when *T* = 0 K or when $\dot{\varepsilon }={\dot{\varepsilon }}_{0}$. According to (14), the applied stress at failure will decrease linearly with temperature and increase linearly with the logarithm of the imposed strain rate. Thus we have a relationship between temperature, strain rate, and stress.

**Figure 11:**
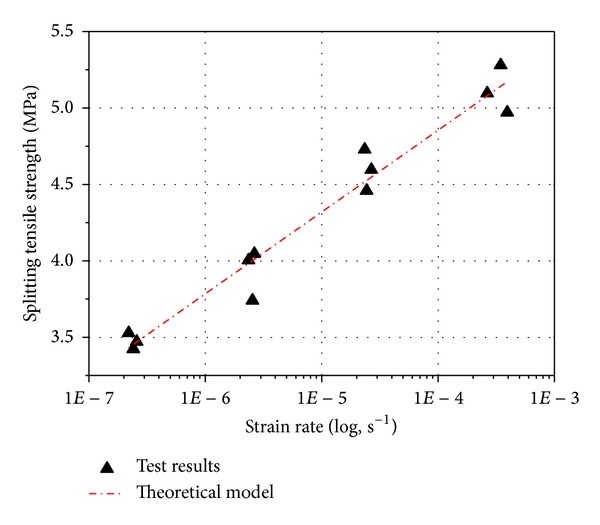
Comparison results between tests and theoretical model.

The constants in this equation are *R* = 1.986 Kcal/mol, *T* = 293 K, *σ*_0_ = 0, *ν* = 2500, and *U*_0_ = 8200 cal/mol.  Figure 11 shows the comparison results between tests and theoretical model in this study; it can be found that the experimental results presented in the preceding show surprisingly good agreement for the entire range in strain rates. Thus, the thermodynamic theory for the splitting tensile strength of concrete satisfactorily describes the experimental findings of strength as affected of strain rates. The coefficient is not influenced by strain rate. Additional experiments to verify the thermodynamic model in compressive and flexural behavior of concrete are underway and will be reported in subsequent papers.

## 5. Conclusions

The following conclusions are drawn from the splitting tensile test results and discussion presented in this paper. The concrete specimens were loaded at strain rates from 10^−7^ to 10^−4^ s^−1^.

1. The fractured surfaces of the specimens became more and more flattened with the increasing strain rate, and an increasing number of aggregates were broken along the fractured surface.
2. With every order of magnitude increase in strain rate, the splitting tensile strength of concrete increases about 15 percent. This trend is nearly linear increase in strength with each order of magnitude increase in strain rate. This trend could be interpreted by combining the subcritical crack growth and fracture process zone: the concept of subcritical crack growth is applicable prior to the peak load; the concept of a larger process zone is applied in the postpeak load region, where the unstable crack propagation commences.
3. The experimental evidence shows also that concrete rate dependence is higher in tension than in compression; this phenomenon could be explained by “Stefan's effect.” A comparison with existing empirical models was performed; the results show that, in the quasistatic and moderate strain rate regime, the existing models often underestimate the experimental results. Hence, more suitable expression for designing calculations should be considered.
4. The thermodynamic theory for the splitting tensile strength of concrete satisfactorily describes the experimental findings of strength as effect of strain rates. The coefficient is not influenced by strain rate. Additional experiments to verify the thermodynamic model in compressive and flexural behavior of concrete are underway and will be reported in subsequent papers.

## Figures and Tables

**Table 1:** Details of mix proportions (in kg per cubic meter of concrete).

Cement	Fly ash	Water	Coarse aggregate		Fine aggregate	Superplasticizer	Air-entraining admixture
			5~20 mm	20~40 mm			
134	57	86	548	390	534	1.43	0.01

**Table 2:** Standard specimens for the Brazilian test.

Standard	Specimen				Bearing strip, *b* (mm)
	Notation	Type	*D* (mm)	*B* (mm)	
ASTM C496	ASTM 150/16	Cylindrical	150	300	25
BS 1881-117	BS_c_150/10	Cylindrical	150	300	15 ± 2
BS 1881-117	BS_q_100/4	Cubical	100	100	4 ± 1
BS 1881-117	BS_q_100/15	Cubical	100	100	15 ± 2
BS 1881-117	BS_q_150/4	Cubical	150	150	6 ± 1
BS 1881-117	BS_q_150/10	Cubical	150	150	15 ± 2

**Table 3:** Summary of strain rate tests.

Specimen number	Loading rate (kN/s)	Time to failure (s)	Maximum load (kN)	Strain rate (s^−1^)	Splitting tensile strength (MPa)	DIF
w101107	0.25	449.68	122.70	2.57 × 10^−7^	3.47	1
w101201		535.03	124.70	2.20 × 10^−7^	3.52	1
w101304		472.37	121.00	2.42 × 10^−7^	3.42	1

w101407	2.5	51.03	143.00	2.64 × 10^−6^	4.04	1.16
w101103		56.66	141.50	2.35 × 10^−6^	4.00	1.15
w100812		48.96	132.20	2.54 × 10^−6^	3.74	1.07

w100807	25	5.72	162.40	2.67 × 10^−6^	4.59	1.32
w101203		6.72	167.10	2.34 × 10^−5^	4.72	1.36
w101804		6.1	157.60	2.43 × 10^−5^	4.45	1.28

w101802	250	0.42	175.70	3.95 × 10^−4^	4.97	1.43
w101206		0.64	180.10	2.65 × 10^−4^	5.09	1.46
w101703		0.51	186.60	3.45 × 10^−4^	5.27	1.51
